# The New Hospital for Women

**Published:** 1890-05-03

**Authors:** 


### Passing Topics.

### THE NEW HOSPITAL FOR WOMEN.

In the completion and opening of the new building of the "New Hospital for Women" we witness a notable illustration of those advances in social development so abundantly associated with the second half of the nineteenth century, and, in the opinion of those who have convinced themselves of the necessity for this institution, and of the larger number who, without having thought much about it, are amiably acquiescent, it will appear, possibly, a startling and almost inhuman proceeding to suggest a doubt of the utility of founding one more impecunious hospital as a charge upon the benevolence experience has shown to be not quite equal to the maintenance of those already in existence, and which contain, as we are frequently assured, a considerable number of beds unoccupied for want of funds.

We admit ungrudgingly that the idea of making provision for the treatment of sick women by qualified persons of their own sex has something to recommend it; that it is precisely the sort of idea calculated to prove attractive to certain sentimental minds, and that the ability and energy with which the undertaking has been carried out are conspicuous. The present result is the erection of a handsome building, well designed for its purpose, and occupying a good position in a leading thoroughfare, where it cannot fail to become a familiar landmark and a standing testimony to the reality of the demand it is calculated to help in creating. If we cannot offer our congratulations to the promoters without *arrière pensée* of any sort it is only because we venture to doubt that their labours have been expended upon a wholly useful object.

We are not disposed to deny, and still less to condemn, the desire on the part of some women for the exclusiveness to be fostered in this institution. We have often inclined to a sympathy for women patients subjected, as they must be if medical science is to be maintained, to observations by youthful students, conceived rather in the interests of education than needful to the treatment of the patient. Yet we have never learned that patients have been commonly distressed on this account, while it is well known in hospital life that a large proportion of women are gratified at the knowledge that their maladies possess interesting and instructive features.

People, whether men or women, are not all constituted alike, and circumstances may cause them to differ radically; yet, the most benign administration would be unequal to making provision which should embrace every individual want, or cater for every individual infirmity. For instance, there are male patients to whom the ministrations of women nurses comprise a more or less painful ordeal transcending anything endured by women at the hands of doctors. Still, we have never heard of a proposal to acknowledge this fact by the erection of a hospital for men, from which all female help should be shut out.

If there be a real demand for the services of this institution such as, sentiment apart, would justify its being, it must be overwhelmed by the multitude of competitors for its favours. Events will show whether the women patients of our great hospitals will desert these institutions solely in order to obtain treatment by persons of their own sex. Faith in women doctors is not very widely disseminated, and women surgeons are scarcely regarded seriously as yet, whatever exceptional women have shown themselves capable of doing. The probability is that the New Hospital for Women will continue to be made use of by the following it has hitherto attracted, some of whom will be drawn thither by real scruples, while others allured for a while by curiosity and a sense of novelty, will fall away in times of grave illness.

Our great schools of medicine may regard the future without misgiving. For some generations at least, they need not fear the flight and emigration of their female subjects. The new hospital is a pioneer which is not likely to move rapidly towards the foundation of new empires. The modest ambitions of its promoters will doubtless be satisfied with minor achievements, and for some time to come they will be concerned to demonstrate that the hospital has been designed in the interests of its patients rather than as an offering to the professional exigencies of the lady doctors.

Agricola.

